# Corrosion Behavior of Copper Foil on PCB Substrates Under Atmospheric Environment in Sichuan-Tibet Region of China

**DOI:** 10.3390/ma17246039

**Published:** 2024-12-10

**Authors:** Xueqing Yang, Haifeng Jin, Dongfang Jia, Jinke Wang, Zhibin Chen, Rui Tian, Shiyao Du, Lingwei Ma

**Affiliations:** 1 National Materials Corrosion and Protection Data Center, University of Science and Technology Beijing, Beijing 100083, China; yang_xueqing1024@163.com (X.Y.); df_jia177@163.com (D.J.); d202110710@xs.ustb.edu.cn (J.W.); chenzhibin0131@163.com (Z.C.); tr19990913@163.com (R.T.); b2405464@ustb.edu.cn (S.D.); 2 Marine Design and Research Institute of China, Shanghai 200011, China; 13120172565@163.com

**Keywords:** printed circuit boards, copper foil, atmospheric corrosion, electrochemistry, Sichuan-Tibet region

## Abstract

Copper foil is widely used in electronic components and devices. This study investigates the corrosion behavior of copper foil on printed circuit boards exposed for one year in a closed atmospheric environment across 22 different sites in the Sichuan-Tibet region. Through electrochemical, SEM/EDS, and XRD analyses, the corrosion behavior of copper foil material across the five selected sites (Meishan, Mangkang, Luding, Batang, and Panzhihua) and the influence of environmental factors were discussed. The results show that copper foils in areas with a large temperature change, higher humidity, and more rainfall exhibit more severe corrosion. The corrosion products primarily form a double-layer structure; the bottom layer consists of relatively stable Cu_2_O, while the type of corrosion product in the upper layer is strongly influenced by the local climatic environment, predominantly containing CuSO_4_, CuSO_4_(OH), and Cu_2_Cl(OH)_3_. In dry areas, copper oxides tend to form, whereas in humid areas, copper sulfides are more likely to grow.

## 1. Introduction

Due to its excellent electrical conductivity, thermal conductivity, ease of processing, and reliable soldering, copper is widely used as an electrical connection carrier for various components and circuits [1,2]. In the electronics field, copper foil is commonly used as a conductive material for the substrate of printed circuit boards (PCBs), serving as the conductive and grounding layers of the circuit board [3]. Copper foil layers, known as copper-clad laminates, are prone to oxidation or electrochemical corrosion in atmospheric environments [4,5]. As electronic circuits and components evolve towards higher density, miniaturization, and higher integration, copper foil on PCBs faces increasing challenges [6]. In complex atmospheric environments, the corrosion of copper foil can affect the performance of electronic devices to varying degrees, such as reducing the conductivity of the copper foil, affecting its electrical performance and reliability, and even causing short or open circuit behavior [7].

Atmospheric corrosion refers to electrochemical corrosion that occurs within a thin condensed water film in an atmospheric environment [8]. The influence of humidity on the condensation phenomenon in the atmospheric environment is evident [9,10]. Starting at approximately 60% relative humidity (RH), a very thin film of moisture forms on the metal surface, leading to the initiation of corrosion, which is known as critical humidity. As the relative humidity increases, the thickness of the water film on the metal surface also increases. Hygroscopic pollutants, such as dust, absorb moisture from the environment, increasing the humidity on the metal surface and accelerating the corrosion process [11,12,13,14,15]. This form of corrosion is ubiquitous. Although corrosion in a closed atmospheric environment is generally less severe than that occurring outdoors, it remains an important research area, particularly in the electronics field, where even minor corrosion damage can cause electronic equipment failure.

Currently, research on the corrosion behavior of copper primarily focuses on outdoor atmospheric exposure environments (such as humid marine and dry areas) and indoor accelerated corrosion tests [16,17,18,19,20]. For example, Mendoza et al. [21] investigated the structure of copper corrosion products in different atmospheric environments in the tropical climate of Cuba, showing that the products were primarily Cu_2_O, Cu_2_Cl(OH)_3_, Cu_4_SO_4_(OH)_6_·2H_2_O, and Cu_4_SO_4_(OH)_6_. Kong et al. [22] conducted a three-year atmospheric corrosion study on pure copper in Turpan, Xinjiang, China, and found that the corrosion product layer formed on the surface of copper could enhance its corrosion resistance, but the protective effect decreased with increasing temperature. Feng et al. [23] conducted outdoor exposure experiments on PCB-Cu in a tropical marine atmosphere and found that long-term exposure led to the formation of a double-layer structure, with a dense oxide layer forming internally and loose corrosion products forming externally. Wu et al. [1] analyzed the corrosion failure of copper wires in outdoor junction boxes of substations, identified Cu_2_Cl(OH)_3_ products in field investigations, and determined that chloride ions and relative humidity are the key factors affecting the corrosion behavior of copper wires. However, there is limited research on the corrosion behavior of copper foil on PCBs in a closed atmospheric environment.

With the accelerated deployment of 5G networks, the construction of communication infrastructure, and the technological advancements in the Sichuan-Tibet region, the performance of copper foil on PCBs is increasingly critical for the longevity of electronic network equipment [24]. The environmental characteristics of the Sichuan-Tibet region, with its high altitude, plateau climate, strong ultraviolet radiation, extreme dryness, and large temperature change, endow higher demands on the corrosion protection of circuit board copper foil. In Tibet, the environment is characterized by long sunshine hours, strong radiation, low temperatures, significant temperature fluctuations, distinct wet and dry seasons, significant nighttime rainfall, relatively dry winters and springs, frequent strong winds, high altitude, and low atmospheric pressure [24]. In contrast, the environment in the Sichuan region is characterized by high precipitation but with dry winter, spring droughts, summer floods, and autumn rains. The annual precipitation distribution is uneven, with about 70% of the rainfall from early June to October each year. Moreover, the pH values of rainfall across various cities in Sichuan fluctuate between 5.09 and 7.54 throughout the year, with acid rain having a pH of 4.82 and a frequency of up to 11.3% [25,26]. To better understand the service differences of copper foil in the Sichuan-Tibet region, accumulating the corrosion data and studying the corrosion behavior of copper foil in typical atmospheric environments across the region is of great significance. Therefore, in this study, we investigate the service differences of copper foil on circuit boards across 22 outdoor exposure sites in the Sichuan-Tibet region, aiming to study the impact of atmospheric corrosion and the environmental characteristics of different sites on the corrosion behavior of copper foil.

## 2. Materials and Methods

### 2.1. Experimental Materials

**Figure 1:**
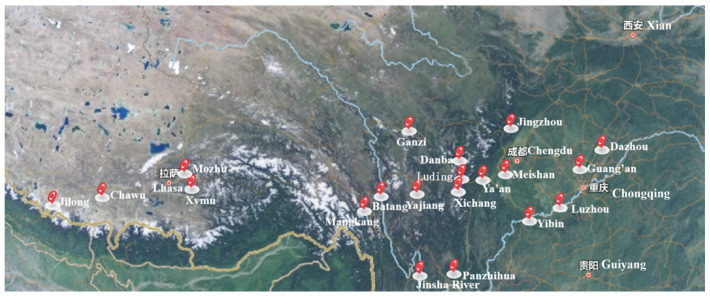
Location maps of different sites.

**Figure 2:**
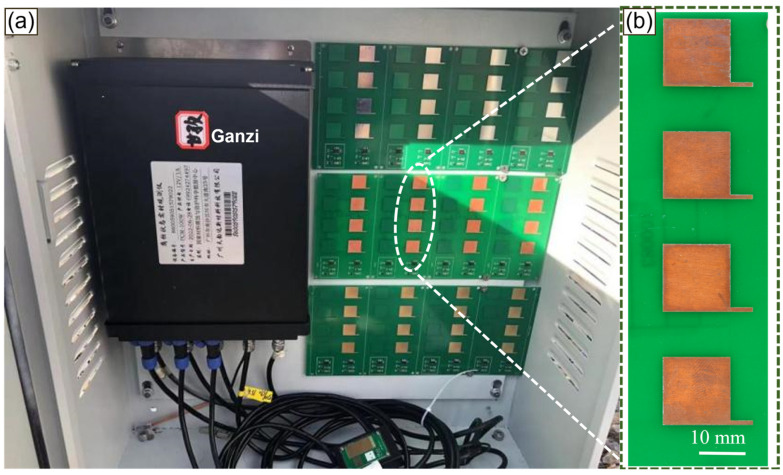
(**a**) Copper foil placement site and (**b**) photos of the circuit board under a closed atmosphere in the Sichuan-Tibet area.

To investigate the service differences of copper foil in the Sichuan-Tibet region, 22 sites were selected (as shown in Figure 1), including Ganzi, Meishan, Chengdu, Luzhou, Xichang, Dazhou, Jinsha River, Panzhihua, Jingzhou, Yajiang, Batang, Guang’an, Danba, Yibin, Ya’an, Luding, Jilong, Mozhu, Lantsang, Xvmu, Mangkang, and Chawu. The latitude and longitude of these sites are provided in Table 1. The copper foil was exposed to a closed atmospheric environment at each site for one year (see Figure 2a) to study the patterns of atmospheric corrosion and the influence of site-specific environmental characteristics on corrosion behavior.

The experimental material, electrolytic copper foil for circuit boards, has a purity exceeding 99.95%, with dimensions of 15 × 15 mm^2^ and a thickness of 35 μm. The macroscopic images of the copper foil are shown in Figure 2b. The base layer is an FR-4 board, composed of epoxy resin and glass fiber cloth laminated together. The exposure period for the samples was from January 2023 to January 2024.

The detailed experimental design is described as follows:

Experimental objectives: To evaluate the atmospheric corrosion resistance of copper foil materials, explore corrosion patterns, and investigate the effects of environmental factors on the performance of copper foil materials.

Experimental materials: Integrated circuit board copper foil.

Exposure environment: Test chambers simulating atmospheric conditions in the Sichuan-Tibet region of China.

Exposure duration: One year.

Environmental data: Temperature, rainfall, and atmospheric pollutants.

Analytical techniques: Scanning electron microscopy (SEM), energy-dispersive X-ray spectroscopy (EDS), and X-ray diffraction (XRD).

Performance analysis: Electrochemical impedance spectroscopy (EIS). For each test, three parallel samples were measured to ensure data reliability.

### 2.2. Experimental Methods

The copper foils were exposed to the closed atmospheric environment of each site in the Sichuan-Tibet region for one year. After the experiment, macroscopic corrosion morphologies were taken using a digital camera. A laser confocal microscope was employed to observe and analyze the microscopic corrosion morphology, and an SEM (ZEISS Sigma 300, Oberkochen, Germany) was used to examine the corrosion products on the sample surface. Additionally, EDS was conducted to analyze the elemental composition of the corrosion products, and XRD (Rigaku SmartLab SE, Tokyo, Japan) was used to analyze the phases of the corrosion products. The XRD scanning rate was set to 2°/min, and the scanning range was 10° to 80°.

**Figure 3:**
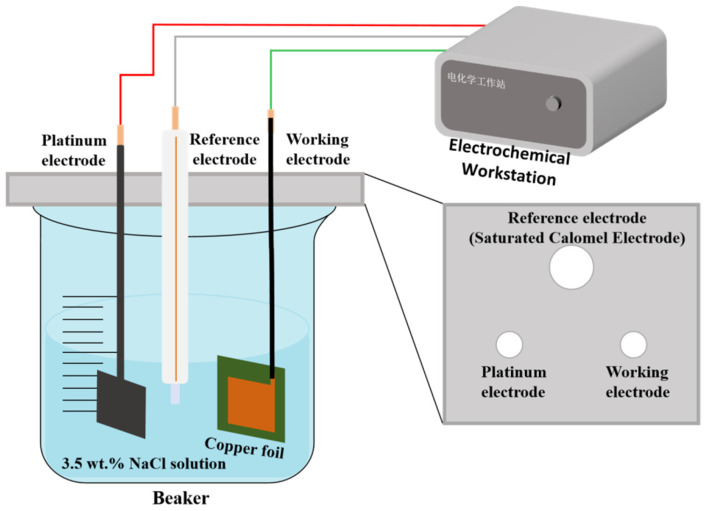
Electrochemical workstation working diagram.

EIS was performed according to the ASTM G106 standard at room temperature using a CHI660E electrochemical workstation. The copper foil served as the working electrode, a platinum sheet was used as the counter electrode, and a saturated calomel electrode (SCE) was employed as the reference electrode. The working area was 15 × 15 mm^2^. Before testing, the open circuit potential (OCP) was stabilized for 30 min. All the EIS measurements were conducted at a frequency range of 10^5^ to 10^−2^ Hz, with a sinusoidal voltage perturbation amplitude of 10 mV relative to the OCP. The electrolyte solution was 3.5 wt.% NaCl solution. For each test, three parallel samples were measured to ensure the reliability of the data. The working process of the electrochemical workstation is shown in Figure 3. The EIS data were fitted using the ZSimpWin 3.6 software.

## 3. Results and Discussion

### 3.1. Macroscopic Corrosion Morphology

**Figure 4:**
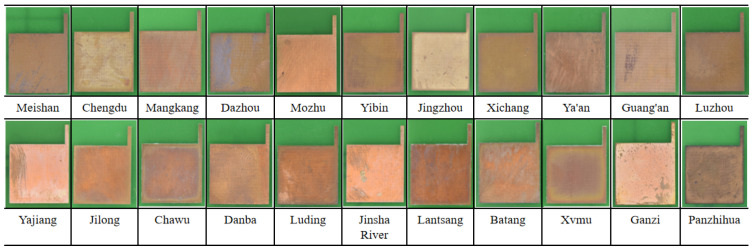
Macroscopic morphology of corrosion of samples after 1-year exposure to a closed atmospheric environment across 22 sites.

Figure 4 shows the macroscopic corrosion morphology of integrated circuit board copper foils after one year of exposure in a closed atmospheric environment across 22 sites. Due to the different climatic conditions, the samples exhibited varying degrees of corrosion across different sites, with noticeable changes in surface color and a loss of metallic luster. The corrosion behavior in Meishan, Mozhu, Mangkang, and Chengdu was relatively mild, followed by Yibin, Luzhou, Xichang, and Jingzhou. The most severe corrosion occurred in Lantsang, Batang, and Panzhihua. For example, the surface of the copper foil in Meishan exhibited slight corrosion scratches, with the surface color remaining close to the original copper color, and the oxide layer was relatively uniform. As the corrosion intensified, such as in Batang, localized areas of the copper foil surface showed an accumulation of white corrosion products, with the sample color primarily reddish-brown. The samples from Panzhihua showed the most severe corrosion, with the brownish-black corrosion products adhering to the base material.

### 3.2. Electrochemical Analysis

**Figure 5:**
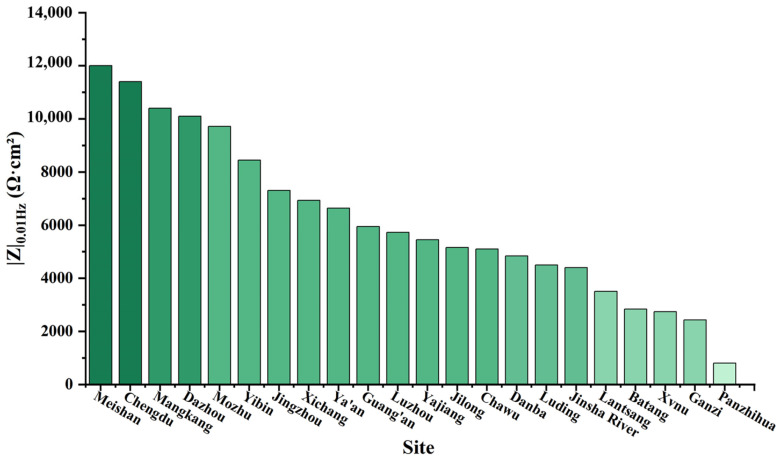
Low-frequency impedance values of copper foil samples placed in a closed atmospheric environment across different stations for 1 year.

The protective effect of the corrosion products formed on the metal substrate is closely related to the nature of these products. Electrochemical testing can effectively assess the corrosion resistance of metals. EIS was used to characterize the corrosion resistance of the copper foil on circuit boards, with the low-frequency impedance at 0.01 Hz (|Z|_0.01Hz_) serving as an important indicator of the sample’s anti-corrosion performance. The low-frequency impedance values were measured after immersing the samples in a 3.5 wt.% NaCl solution for 3 days, as shown in Figure 5. The results indicate that the |Z|_0.01Hz_ values of the samples from Meishan, Chengdu, Mozhu, and Dazhou were relatively high, reaching up to 12,000 Ω·cm^2^. The |Z|_0.01Hz_ values from the Luding, Danba, Luzhou, and Yajiang samples were moderate, while those from Batang and Panzhihua were the lowest, falling below 1000 Ω·cm^2^. Based on the macroscopic morphology and low-frequency impedance data, samples from Meishan, Mangkang, Luding, Batang, and Panzhihua were selected for further investigation.

**Figure 6:**
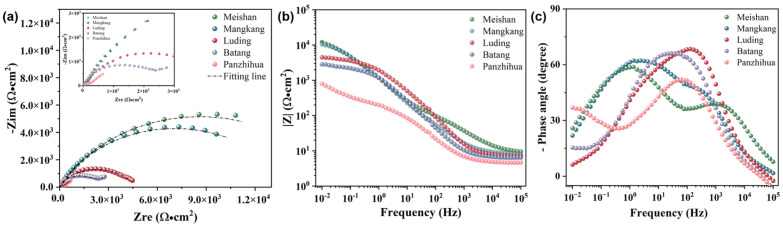
(**a**) Nyquist diagram, (**b**) Bode-impedance modulus plot, and (**c**) Bode-phase angle plot of the samples after the closed atmospheric environment exposure experiment across 5 sites.

Figure 6 shows the EIS data of the copper foil exposed for one year at five stations—Meishan, Mangkang, Luding, Batang, and Panzhihua. In the Nyquist plot (Figure 6a), all samples exhibit a single capacitive arc. However, the impedance of the samples from Panzhihua displays a diffusion tail in the low-frequency region, corresponding to Warburg impedance. This likely includes the diffusion of soluble metal ions from the sample surface to the anode in the solution, as well as the diffusion of dissolved oxygen toward the cathode in the opposite direction. Among the five sites, the Meishan samples exhibited the largest capacitive arc radius, indicating the best corrosion resistance, while the Panzhihua samples showed the smallest capacitive arc radius with the most severe corrosion behavior. In the Bode-impedance modulus plot (Figure 6b), the |Z|_0.01Hz_ values for the Meishan and Mangkang samples were similar, at 12,000 Ω·cm^2^ and 10,400 Ω·cm^2^, respectively. The copper foil exposed at Luding and Batang followed, with values of 4500 Ω·cm^2^ and 2840 Ω·cm^2^, respectively. The sample from Panzhihua had the smallest |Z|_0.01Hz_ value, at only 802 Ω·cm^2^, indicating the worst corrosion resistance. In the phase angle plot (Figure 6c), two time constants were observed on the sample surfaces, corresponding to the corrosion product film and the interface corrosion reaction. The width of the phase angle change curve reflects the tendency of the sample to corrode; a wider phase angle frequency range indicates better corrosion resistance. Overall, the |Z|_0.01Hz_ values decreased in the following order: Meishan, Mangkang, Luding, Batang, and Panzhihua, while the phase angle range narrowed, indicating a gradual decline in the corrosion resistance of the sample surfaces across these five sites.

**Figure 7:**
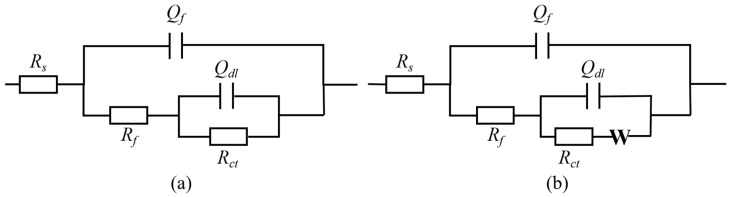
Equivalent circuit used to fit EIS data for copper in the following: (**a**) Meishan, Mangkang, Luding; and (**b**) Batang, Panzhihua.

As shown in Figure 7, two equivalent circuits were used to fit the EIS results. The equivalent circuit shown in Figure 7a was employed to fit the EIS results of samples from the Meishan, Markam, and Luding sites. Meanwhile, the equivalent circuit in Figure 7b was used for fitting the EIS data from the Batang and Panzhihua sites due to the apparent diffusion tail observed in the low-frequency region, indicating the occurrence of surface diffusion processes. In the equivalent circuits, *R*_s_ represents the electrolyte resistance, *R*_ct_ represents the charge transfer resistance on the metal substrate, *R*_f_ represents the resistance of the surface corrosion product layer, *Q*_dl_ and *Q*_f_ are, respectively, the double-layer capacitance and the capacitance of the corrosion product layer, and W is the Warburg impedance in the low-frequency region. When using the equivalent circuits for fitting, the chi-square values all reach the order of magnitude of 10^−3^, indicating the applicability of the equivalent circuits. The fitting results of the EIS data are shown in Table 2. The total resistance *R*_t_ is defined as the sum of the *R*_ct_ and *R*_f_, which can be used to evaluate the overall corrosion resistance of the samples. The fitting results of *R*_f_, *R*_ct_, and *R*_f_ show a consistent variation trend. Specifically, the resistance values of the samples across the five sites of Meishan, Markam, Luding, Batang, and Panzhihua gradually decrease.

### 3.3. Microscopic Corrosion Morphology and Composition Analysis

**Figure 8:**
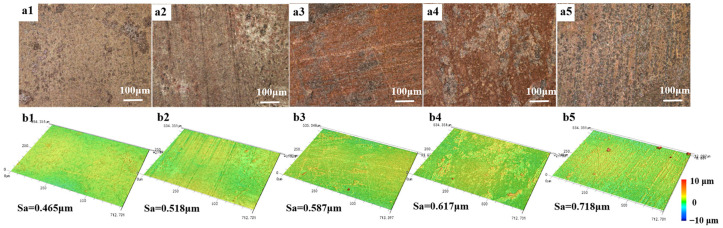
The micro-topography and surface roughness of samples placed in a closed atmospheric environment across different stations for 1 year: (**a1**,**b1**) Meishan, (**a2**,**b2**) Mangkang, (**a3**,**b3**) Luding, (**a4**,**b4**) Batang, and (**a5**,**b5**) Panzhihua.

To assess the degree of corrosion on the sample surface, Figure 8 presents the surface morphology and 3D confocal images of the samples after one year of exposure at Meishan, Mangkang, Luding, Batang, and Panzhihua. Among these sites, the copper foil surface in Meishan exhibited no significant scratches, with only a few irregular spot-like protrusions of corrosion products and the lowest surface roughness. This is likely due to the relatively mild environmental conditions, which helped maintain its high corrosion resistance and significantly slowed the progression of localized corrosion. In Mangkang, noticeable scratches appeared on the sample surface, with reddish-brown corrosion products accumulating in the scratches, identified as copper oxides [16,27]. In Luding, the sample surface showed prominent scratches, accompanied by large patches of reddish-brown and black, irregularly shaped corrosion products. The roughness was significantly higher in areas containing black corrosion products. The corrosion became more severe in Batang, where the corrosion product layer exhibited a distinct double-layer structure: the bottom layer comprised reddish-brown corrosion products, while the upper layer featured white stripe-like corrosion products [28]. The most severe corrosion was observed in Panzhihua, which has the highest average annual temperature and the lowest rainfall. The sample surface displayed complex and unevenly distributed corrosion products, including irregular black corrosion product protrusions, primarily attributed to the further oxidation of copper, forming CuO [29].

**Figure 9:**
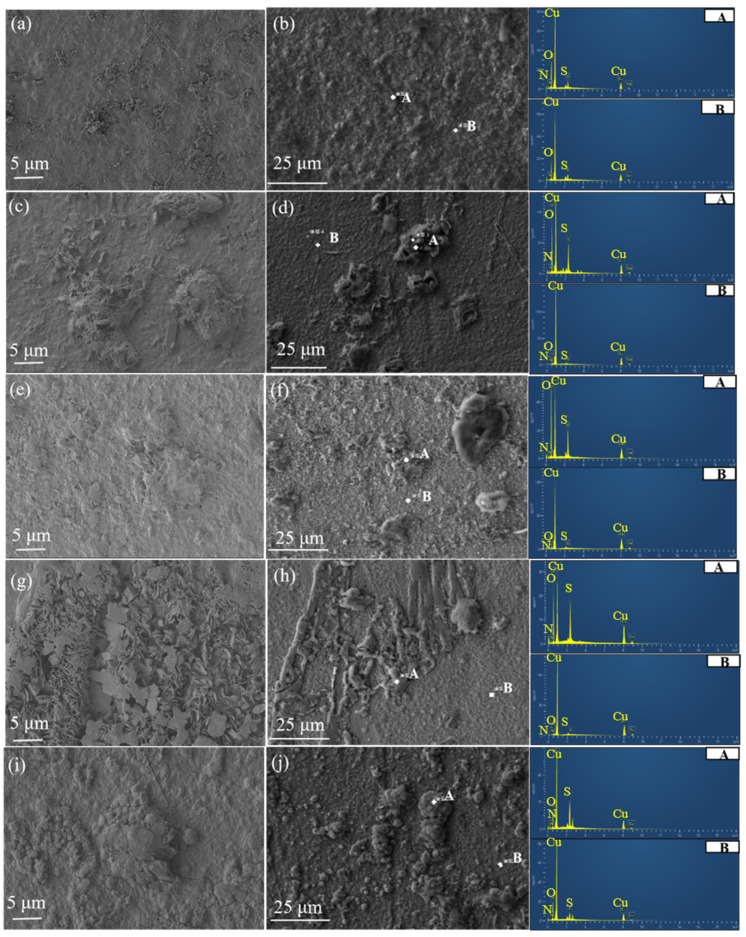
SEM images of corrosion products and EDS images of corresponding corrosion products at point A and point B after samples were placed in a closed atmospheric environment across different stations for 1 year: (**a**,**b**) Meishan, (**c**,**d**) Mangkang, (**e**,**f**) Luding, (**g**,**h**) Batang, and (**i**,**j**) Panzhihua.

Figure 9 shows the SEM images of corrosion products and the corresponding EDS spectra of samples. In the EDS spectra, region A corresponds to the thicker corrosion product protrusions, while region B represents corrosion products closer to the copper substrate. In Figure 9a,b, the morphology of the corrosion product of the Meishan sample appeared as layered, dot-like protrusions. The similar elemental composition in regions A and B indicates relatively uniform corrosion with low sulfur content. The corrosion products were primarily composed of copper oxides [30]. In Figure 9c,d, the SEM images of the Mangkang sample revealed significant compositional differences between regions A and B, indicating a double-layer structure in the corrosion products. The first layer of corrosion products in region B was relatively thin, mainly consisting of copper oxides, while the second layer in region A was thicker and loosely distributed, primarily consisting of copper oxides and sulfides. In Figure 9e,f, the sulfur and oxygen contents in region A of the sample from Luding were significantly higher than in region B, indicating that the corrosion products in region A were likely composed of both copper oxides and sulfides. In Figure 9g,h, the oxygen content in the corrosion products of the Batang sample was similar to that of the samples from Luding and Mangkang, but the sulfur content was relatively high, indicating that the corrosion products formed among these three sites had similar structures. However, the Batang sample had more sulfide content in the second layer of corrosion products. In Figure 9i,j, the sample from Panzhihua showed several surface cracks, which reduced the adhesion of the oxide layer to the substrate, accelerating the corrosion process. The EDS analysis revealed that the corrosion products primarily consisted of oxides with a small amount of sulfides. Overall, the formation of the loosely distributed corrosion products on the sample surfaces involved the interweaving of lamellar products, which eventually formed tightly connected, cluster-like corrosion products.

**Figure 10:**
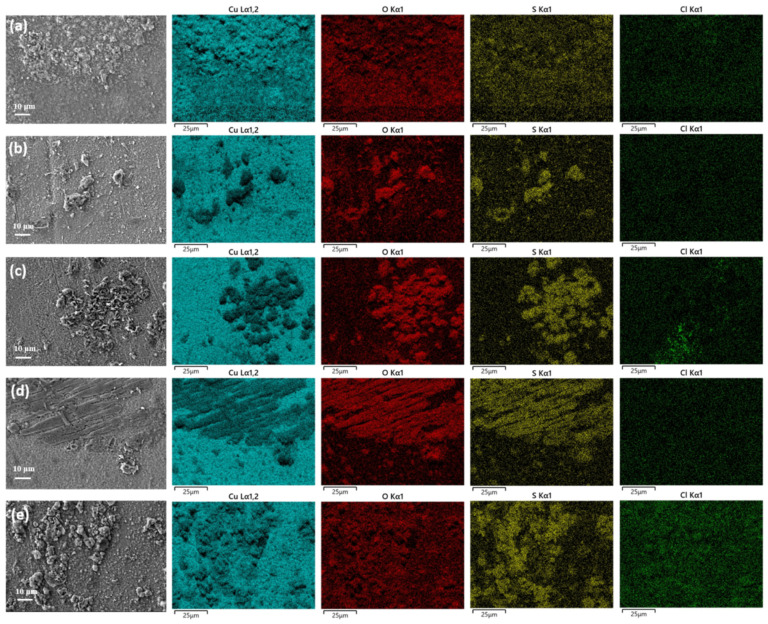
Elemental distribution of corrosion products on the surface of copper foil samples: (**a**) Meishan, (**b**) Mangkang, (**c**) Luding, (**d**) Batang, and (**e**) Panzhihua.

Figure 10 shows the elemental distribution on the surface of corrosion products of copper foil under a closed atmospheric environment across five typical sites. It can be seen that sulfur elements mainly exist in the raised area of corrosion products, while oxygen elements are primarily present in the underlying corrosion products. Among the sites, the Meishan sample exhibits the lowest sulfur content with a thin layer of corrosion products. Moreover, the corrosion products at Meishan and Panzhihua exhibit a more uniform distribution of oxygen elements. The overall elemental distribution results are consistent with the EDS results discussed earlier.

**Figure 11:**
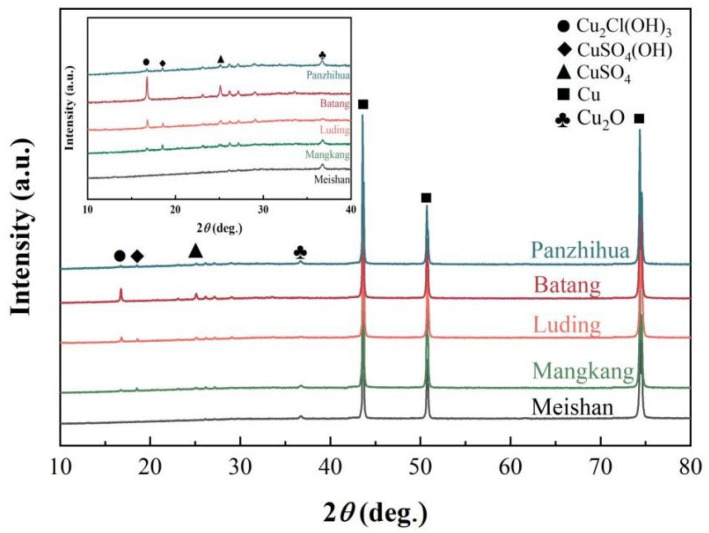
XRD patterns of corrosion products on copper foil surface across different sites.

Figure 11 shows the XRD test results of corrosion products on copper foils. Combining the results mentioned above, the main elements of the rust layer on the sample surface were Cu, O, and S, and the corrosion products mainly consisted of copper oxides and sulfides. The XRD results revealed peaks corresponding to copper oxides (Cu_2_O and CuO), copper sulfides (CuSO_4_(OH) and CuSO_4_), and CuCl(OH)_3_, respectively. The corrosion products on the Meishan samples consisted exclusively of Cu_2_O. For the samples from Luding and Batang, distinct peaks of sulfides and chlorides, including CuSO_4_, CuSO_4_(OH), and Cu_2_Cl(OH)_3_, were observed, corresponding to the white corrosion products identified in the macroscopic morphology. In contrast, the corrosion products on the Panzhihua samples primarily consisted of copper oxides, with no significant sulfides observed.

### 3.4. Discussion of Environmental Factors

**Figure 12:**
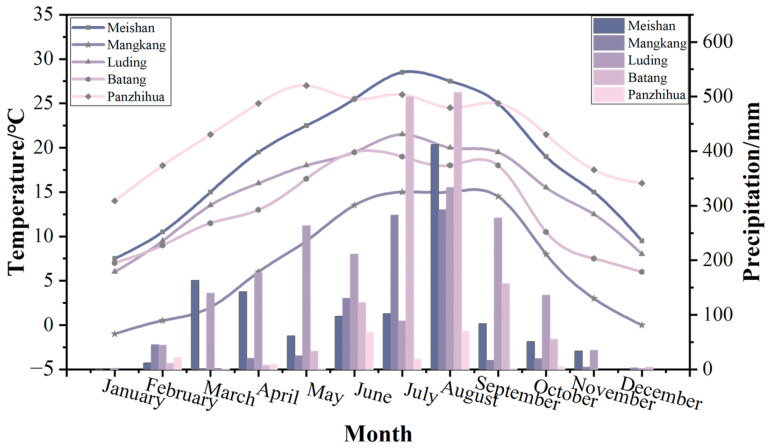
Monthly mean temperature and monthly precipitation across five stations in 2023.

**Figure 13:**
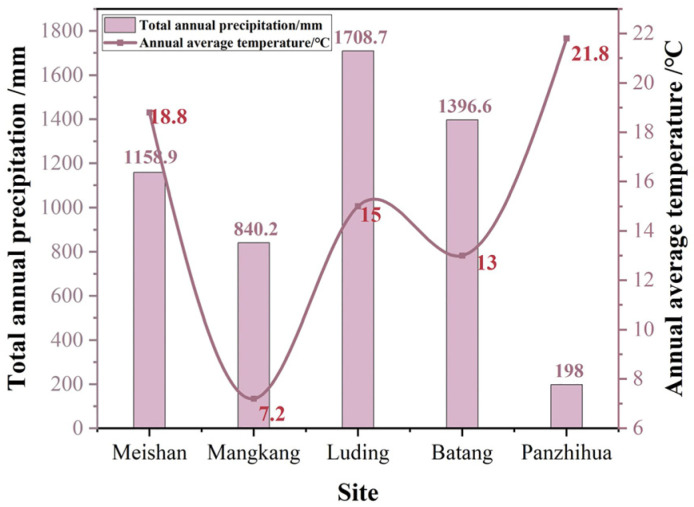
Annual mean temperature and annual precipitation across five stations in 2023.

The degree of corrosion of copper foil samples is strongly related to the environmental factors of the region where they were exposed to the atmosphere, such as temperature, salt dust, relative humidity, and pH values [5,31]. Figure 12 and Figure 13 focus on the meteorological data for the year 2023 in the five regions of Meishan, Mangkang, Luding, Batang, and Panzhihua. Figure 12 shows the monthly average temperature and total precipitation at the five stations, while Figure 13 presents the annual average temperature and total precipitation among the five sites. Meishan is located in the western part of the Sichuan Basin and features a subtropical humid monsoon climate with four distinct seasons. The monthly temperatures exhibit an unimodal variation, ranging from an average of 6.3–7.5 °C in January to 25.7–26.7 °C in July. The annual precipitation exceeds 1000 mm. Mangkang, located in the southeastern part of the Qinghai-Tibet Plateau, experiences the lowest temperatures, with an average temperature of −1 °C in January and 15 °C in July, and an annual average temperature of 7.2 °C. Precipitation is unevenly distributed throughout the year, resulting in mild, humid summers and cold, dry winters, with a frost period lasting up to 270 days. Luding is a typical alpine gorge region, with an annual average temperature of 15 °C, annual precipitation of approximately 1708 mm, and 332 frost-free days annually. In Batang, precipitation increases significantly in July and August, with an annual average temperature of 13 °C. Panzhihua, characterized by the highest temperature and lowest rainfall, has a typical dry–hot valley climate featuring a long summer, no winter, extended sunshine hours, and intense solar radiation.

Based on the analysis of the composition and morphology of corrosion products at the five typical stations in the Sichuan-Tibet region, temperature and relative humidity are crucial factors affecting the corrosion of copper foil in a closed atmospheric environment. The samples in areas with higher rainfall and larger variations in temperature and humidity exhibited poorer stability. Additionally, SO_2_ is one of the most severe atmospheric corrosion gases, especially when SO_2_ and water vapor co-exist, leading to severe corrosion. This is because when dust and atmospheric pollutants deposit on the copper foil surface, the critical relative humidity of the copper foil surface is significantly reduced, making it easier for an electrolyte film to form and cause corrosion on the copper foil. In terms of corrosion product composition, copper oxides tend to form in dry–hot regions, while copper sulfides are more likely to form in humid–hot areas. For example, in Batang and Luding, where precipitation and relative humidity are high, the adsorption of SO_2_ gas from the air promotes the formation of sulfides, thereby exacerbating corrosion. In Panzhihua, despite the highest temperatures, the corrosion products mainly consisted of oxides, with very few sulfides, primarily due to the dry–hot climate and low relative humidity. Furthermore, in regions with adsorptive sea salt ions in the atmosphere, the corrosion products on copper foil may include basic copper chlorides [32].

### 3.5. Discussion of Corrosion Mechanism

**Figure 14:**
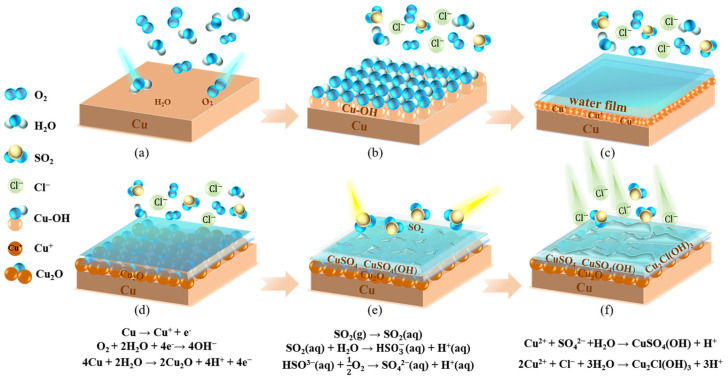
Schematic diagram of electrochemical corrosion of circuit board copper foil.

The corrosion of copper foil in an atmospheric environment primarily follows an electrochemical corrosion mechanism consisting of a cathode, anode, electronic conductor, and ion conductor (electrolyte on the metal surface). This process can be considered a short-circuited galvanic cell, primarily comprising the anodic reaction of copper dissolution with electron loss and the cathodic reaction of oxygen reduction or water decomposition within the liquid film. The atmospheric factors affecting corrosion in these five sites are mainly oxygen, water, and sulfur dioxide (as shown in Figure 14a). During the corrosion process, the dissolved copper ions react with the atmospheric components to form multiple phases, including Cu^+^ and Cu^2+^ valence states [33]. The main reactions are as follows:

When exposed to the atmosphere, the copper foil on the circuit board rapidly adsorbs an initial layer of water, resulting in the formation of a copper and hydroxyl group adsorption pair (Cu-OH), as shown in Figure 14b. Subsequently, atmospheric water vapor continues to adsorb on the metal surface, leading to the development of a thin electrolyte film, as shown in Figure 14c, where the corrosion rate remains relatively slow. Once the atmospheric humidity exceeds the critical relative humidity, a continuous thin water film forms on the copper foil surface, significantly increasing the corrosion rate. Beneath this thin electrolyte film, the copper anodic dissolution reaction occurs first, forming ${Cu}^{+}$/${Cu}^{2+}$, while the cathodic reaction mainly involves oxygen reduction or water decomposition, precipitating Cu_2_O on the copper surface, as shown in Figure 14d.

(1) $$Cu\to {Cu}^{+}+e^{-}$$

(2) $$O_{2}+2H_{2}O+4e^{-}\to 4OH^{-}$$

(3) $$4Cu+2H_{2}O\to 2{Cu}_{2}O+4H^{+}+4e^{-}$$

Subsequently, weak anodic reactions occur simultaneously on the cathode plate, forming a CuO product layer. In humid air, CuO readily reacts with SO_2_ to produce sulfides.

(4) $$2Cu_{2}O+O_{2}\to 4CuO$$

When the adsorptive salt ions, dust, atmospheric pollutants, and corrosion products deposit on the copper foil surface, the critical relative humidity of the copper surface decreases significantly. This promotes the formation of an electrolyte film, further accelerating copper foil corrosion. In regions with higher humidity, the electrolyte film on the copper foil surface is relatively thicker, facilitating the dissolution of SO_2_ from the atmosphere. The dissolution process of SO_2_ is shown in Figure 14e, with the reactions as follows:

(5) $$SO_{2}\left(g\right)\to SO_{2}\left(aq\right)$$

(6) $$SO_{2}\left(aq\right)+H_{2}O\to HSO_{3}^{-}\left(aq\right)+H^{+}\left(aq\right)$$

(7) $$HSO_{3}^{-}\left(aq\right)+\frac{1}{2}O_{2}\to SO_{4}^{2-}\left(aq\right)+H^{+}\left(aq\right)$$

${HSO}_{3}^{-}$, $SO_{4}^{2-}$, $H^{+}$, and SO_2_·nH_2_O co-exist in the water film on the copper foil surface, making the water film acidic and accelerating copper corrosion. Therefore, in acidic electrolytes, Cu_2_O in areas with poorer local stability is damaged [34], and the surface oxide film is destroyed, becoming the anodic region of the corrosion microcell and further corroding and dissolving to form CuSO_4_ and CuSO_4_(OH):

(8) $$Cu_{2}O+2H^{+}\to Cu+Cu^{2+}+H_{2}O$$

(9) $$Cu^{2+}+SO_{4}^{2-}+H_{2}O\to CuSO_{4}\left(OH\right)+H^{+}$$

When ${Cl}^{-}$ is present in the environment, the copper ions generated by the dissolution of Cu_2_O react with it to form Cu_2_Cl(OH)_3_:

(10) $$2{Cu}^{2+}+{Cl}^{-}+3H_{2}O\to {Cu}_{2}Cl{\left(OH\right)}_{3}+3H^{+}$$

When both SO_2_ and ${Cl}^{-}$ exist on the copper foil surface, the atmospheric corrosion of copper occurs, as shown in Figure 14f. On the one hand, the presence of sea salt ions can increase the conductivity of the film; on the other hand, SO_2_ dissolved in the film reduces its pH, and these two effects function together to enhance the corrosivity of the film, thereby accelerating the corrosion process of the copper foil.

Based on the above discussion and analysis, the corrosion process of copper foil can be summarized as follows: Initially, Cu_2_O forms preferentially, making the copper foil relatively stable with a low corrosion rate, primarily manifesting as localized corrosion. Over time, Cu_2_O oxidizes to CuO, but due to the deposition of atmospheric pollutants and particles, CuO can quickly react with SO_2_ in humid air to form sulfides, while the adhesion of Cu_2_O to the substrate weakens, reducing its protective effect. When both the atmospheric pollutants, SO_2_ and sea salt ions, deposit on the sample surface, products such as Cu_2_Cl(OH)_3_, CuSO_4_, and CuSO_4_(OH) form on the copper foil surface. These corrosion products are often loose and unevenly distributed, and the Cu_2_O corrosion product layer adjacent to the substrate cracks, causing instability in the copper foil substrate and accelerating corrosion.

## 4. Conclusions

This study analyzed the results of one-year exposure experiments conducted in a closed atmospheric environment across five typical sites in the Sichuan-Tibet region on copper foil.

(1) Among the five regions, the corrosion product layer on the copper foil surface in Meishan was denser and more uniform, and the type of corrosion product was relatively single. While in Mangkang, Luding, Batang, and Panzhihua, the corrosion products were loosely distributed and the surface roughness of the samples increased successively.

(2) The composition analysis of the corrosion products revealed that Cu_2_O was the main corrosion product. A dense and uniform Cu_2_O product layer provided potential protection to the substrate. Subsequently, the deposition of pollutants and particles in the humid atmosphere caused the destruction of Cu_2_O in less stable areas, leading to the breakdown of the surface oxide film and the formation of anodic regions in corrosion microcells. This resulted in further corrosion and dissolution, forming sulfides such as CuSO_4_ and CuSO_4_(OH). Additionally, the presence of Cu_2_Cl(OH)_3_ in the corrosion products from Luding and Batang suggests the existence of Cl^−^ in the atmosphere at these sites. In Panzhihua, the corrosion products primarily consisted of Cu_2_O, with a very small amount of CuO, which quickly reacted with SO_2_ in humid air to generate sulfides.

(3) The presence of both SO_2_ and water vapor resulted in severe corrosion. The samples located in areas with higher rainfall and greater temperature and humidity fluctuations demonstrated reduced stability. The dry–hot regions tend to form copper oxides, whereas the humid-hot regions facilitate the formation of copper sulfides.

In summary, the corrosion of copper foil is susceptible to environmental factors such as air, moisture, acids, and bases, leading to loss of luster and spot formation and significantly affecting its service life and aesthetics. Therefore, this study offers a theoretical basis for enhancing the service life and performance of copper foil in the Sichuan-Tibet region.

## Figures and Tables

**Table 1:** Latitude and longitude of different sites.

Site	Longitude and Latitude	Site	Longitude and Latitude
Meishan	N 29°24′ E 102°49′	Yajiang	N 29°03′ E 100°19′
Chengdu	N 30°05′ E 102°54′	Jilong	N 28°03′ E 84°35′
Mangkang	N 28°37′ E 98°00′	Chawu	N 28°47′ E 87°24′
Dazhou	N 30°19′ E 106°39′	Danba	N 30°24′ E 101°17′
Mozhu	N 29°50′ E 91°44′	Luding	N 29°54′ E 101°46′
Yibin	N 27°50′ E 103°36′	Jinsha River	N 26°32′ E 102°51′
Jingzhou	N 31°25′ E 102°49′	Lantsang	N 22°45′ E 100°57′
Xichang	N 27°32′ E 101°46′	Batang	N 28°46′ E 98°58′
Ya’an	N 28°51′ E 101°55′	Xvmu	N 27°98′ E 85°98′
Guang’an	N 30°01′ E 105°56′	Ganzi	N 27°58′ E 97°22′
Luzhou	N 27°39′ E 105°08′	Panzhihua	N 26°05′ E 101°08′

**Table 2:** EIS fitting results of sample surfaces across different sites.

Sites	R_s_ (Ω·cm^2^)	Y_f_ (S·s^n^·cm^−2^)	n_f_	R_f_ (Ω·cm^2^)	Y_dl_ (S·s^n^·cm^−2^)	n_dl_	R_ct_ (Ω·cm^2^)	Y_W_ (S·s^0.5^·cm^−2^)	R_t_ (Ω·cm^2^)
Meishan	8.43	1.05 × 10^−4^	0.64	1.62 × 10^3^	1.45 × 10^−4^	0.73	1.70 × 10^4^	-	18,620
Mangkang	7.90	2.23 × 10^−4^	0.68	4.75 × 10^2^	1.42 × 10^−5^	1.00	1.38 × 10^4^	-	14,275
Luding	7.30	3.90 × 10^−5^	0.80	5.34 × 10^2^	1.23 × 10^−4^	0.53	4.31 × 10^3^	-	4844
Batang	6.46	1.52 × 10^−5^	1.00	4.57 × 10^0^	1.41 × 10^−4^	0.73	2.29 × 10^3^	3.95 × 10^−3^	2294.57
Panzhihua	6.04	5.89 × 10^−4^	0.69	5.13 × 10^2^	3.79 × 10^−3^	0.62	1.71 × 10^3^	3.22 × 10^−3^	2223

## Data Availability

The original contributions presented in this study are included in the article. Further inquiries can be directed to the corresponding authors.
